# Herbaceous plant species invading natural areas tend to have stronger adaptive root foraging than other naturalized species

**DOI:** 10.3389/fpls.2015.00273

**Published:** 2015-04-27

**Authors:** Lidewij H. Keser, Eric J. W. Visser, Wayne Dawson, Yao-Bin Song, Fei-Hai Yu, Markus Fischer, Ming Dong, Mark van Kleunen

**Affiliations:** ^1^Ecology, Department of Biology, University of KonstanzKonstanz, Germany; ^2^Department of Biology, Institute of Plant Sciences, University of BernBern, Switzerland; ^3^Department of Experimental Plant Ecology, Institute for Water and Wetland Research, Radboud University NijmegenNijmegen, Netherlands; ^4^State Key Laboratory of Vegetation and Environmental Change, Institute of Botany, Chinese Academy of SciencesBeijing, China; ^5^School of Nature Conservation, Beijing Forestry UniversityBeijing, China

**Keywords:** invasion ecology, multi-species comparison, nutrient heterogeneity, phenotypic plasticity, pre-adaptation, root morphology

## Abstract

Although plastic root-foraging responses are thought to be adaptive, as they may optimize nutrient capture of plants, this has rarely been tested. We investigated whether nutrient-foraging responses are adaptive, and whether they pre-adapt alien species to become natural-area invaders. We grew 12 pairs of congeneric species (i.e., 24 species) native to Europe in heterogeneous and homogeneous nutrient environments, and compared their foraging responses and performance. One species in each pair is a USA natural-area invader, and the other one is not. Within species, individuals with strong foraging responses, measured as plasticity in root diameter and specific root length, had a higher biomass. Among species, the ones with strong foraging responses, measured as plasticity in root length and root biomass, had a higher biomass. Our results therefore suggest that root foraging is an adaptive trait. Invasive species showed significantly stronger root-foraging responses than non-invasive species when measured as root diameter. Biomass accumulation was decreased in the heterogeneous vs. the homogeneous environment. In aboveground, but not belowground and total biomass, this decrease was smaller in invasive than in non-invasive species. Our results show that strong plastic root-foraging responses are adaptive, and suggest that it might aid in pre-adapting species to becoming natural-area invaders.

## Introduction

Soil nutrients are generally patchily distributed, frequently at scales as small as a few centimeters (Hodge, 2004). As a result, different parts of a single plant may experience different nutrient conditions. They can respond to this heterogeneity by differentiating their root growth and development between nutrient-poor vs. nutrient-rich soil patches (de Kroon et al., 2009). These plastic root-foraging responses are thought to enable plants to optimize nutrient capture, and increase plant performance (Robinson et al., 1999). Therefore, the capacity for a strong root-foraging response when growing in heterogeneous soil may be expected to be positively correlated to species success or invasiveness. However, it is not known whether or to what extent this is the case.

Many plant species have been introduced to new regions. Some of these species have been able to sustain stable populations (have become naturalized), and some of those have formed new populations and spread rapidly (have become invasive) (Williamson and Fitter, 1996). Most invasions start in anthropogenic environments, and most alien species stay there, but a small subset of alien species manages to also invade natural habitats (Richardson et al., 2000). Frequently, this happens after disturbance events, as predicted by the fluctuating-resources hypothesis (Davis et al., 2000). Few, if any, studies have tested what distinguishes these natural-area invaders from other naturalized alien species. In natural habitats, irrespective of whether they are more or less heterogeneous than anthropogenic environments, alien plants are likely to experience stronger competition from resident species. Under such conditions, it may be especially important to be able to rapidly find and exploit high resource patches (Robinson et al., 1999; Parepa et al., 2013). We therefore expect that successful natural-area invaders have stronger root-foraging responses than unsuccessful ones.

Invasiveness of species is partly determined by their traits (Pyšek and Richardson, 2007; van Kleunen et al., 2010b). Phenotypic plasticity—the change in the expressed phenotype of a genotype as a function of the environment (Bradshaw, 1965)—is frequently mentioned as a trait that potentially promotes invasiveness (Baker, 1965; Richards et al., 2006; Hulme, 2008). Plastic species could express optimal phenotypes under different growing conditions, and this increased environmental tolerance could also allow them to grow in novel environments. In other words, a high plasticity of species in their native ranges could pre-adapt them to the novel environments that they may encounter in their non-native ranges. Some studies found support for a relationship between plasticity and invasiveness (Davidson et al., 2011; Dawson et al., 2012a,b; Keser et al., 2014), but others did not (Schlaepfer et al., 2010; Palacio-López and Gianoli, 2011; van Kleunen et al., 2011). These discrepancies could partly reflect that plasticity of a trait in response to a certain environmental variable is not necessarily adaptive (i.e., does not necessarily increase fitness; van Kleunen and Fischer, 2005). Because plastic changes in root morphology may enhance resource acquisition and thereby performance of the plant, root foraging is likely to be an example of adaptive phenotypic plasticity. Surprisingly, however, it has rarely been tested explicitly whether root foraging increases performance (but see Wang et al., 2013).

To test whether root foraging is adaptive, and whether it generally pre-adapts plant species to invade natural areas, we conducted a multi-species greenhouse experiment. In this experiment, we compared the effect of soil heterogeneity on root morphology and on plant performance of native European plant species differing in their invasion success in natural areas in the USA. We used 24 plant species subdivided into 12 congeneric species pairs from eight plant families, all of which have been introduced to and naturalized in North America. Within each pair, one species is listed as a natural-area invader and the other is not. We grew all species in homogeneous and heterogeneous nutrient environments, and assessed (1) the morphological root-foraging response (root length, root diameter, root biomass, and specific root length) of plants growing in a heterogeneous environment with nutrient-rich and nutrient-poor patches, and (2) the effect of nutrient heterogeneity on the production of aboveground, belowground and total biomass (as a proxy for plant performance) of the plants.

We addressed the following three questions: Are plastic root-foraging responses adaptive in heterogeneous soils? Are European herbaceous plant species pre-adapted to invade natural areas in North America through strong root-foraging responses in heterogeneous soils? And do plant species that are invasive in natural areas experience a more positive or a less negative effect of soil heterogeneity on plant performance compared to species that do not invade natural areas?

## Material and methods

### Species selection and pre-cultivation

We selected a total of 12 congeneric pairs of herbaceous species from eight plant families. All 24 species are native to Europe, and have become naturalized in North America (Online Appendix I). In this experiment, we were interested in the distinction between plant species that do or do not manage to invade natural areas in the USA. To decide whether the species are natural-area invaders or not, we used The Invasive Plant Atlas of the United States (www.invasiveplantatlas.org), which is a comprehensive compilation of alien plant species that invade natural areas in the US. These natural areas do not include agricultural land or other heavily anthropogenic sites. Within each species pair, one species is listed in this atlas as a natural-area invader in at least four USA states, and the other one is not listed as such in any USA state (Online Appendix I). From here on, we refer to the first group as invasive species and to the second group as non-invasive species. It could be that some of our non-invasive species are no natural-area invaders because they did not disseminate into such habitats yet (van Kleunen et al., 2014b). However, as also our non-invasive species are already widely naturalized in North America (see Online Appendix I), we think that this explanation is quite unlikely.

Because several studies have found a link between relative growth rate and the strength of the foraging response of plant species (see references in Kembel and Cahill, 2005), and others between relative growth rate and invasiveness (Grotkopp and Rejmanek, 2007; Dawson et al., 2011), we reduced this potential confounding factor by balancing the invasive and non-invasive species in each pair with respect to the average plant size. Average height was calculated by averaging the minimum and the maximum height of the species as listed in Rothmaler et al. (2005) (Online Appendix I; size difference between invasive and non-invasive species in the species pairs tested with a paired *t*-test: *t* = 0, *p* = 1).

We ordered seeds of our species from botanical gardens throughout Europe and from commercial companies selling wild-collected seeds (see Online Appendix II). Pre-cultivation and the experiment took place in the botanical garden of the University of Konstanz, Germany (N: 47°69′19.56″, E: 9°17′78.42″). For each of the 24 species, we mixed the seeds from the different suppliers. On the 14th of June 2011, we sowed the seeds in trays filled with a 1:1 mixture of sand and fine vermiculite. We first put the trays in a cold room (4°C, 16 h of light per day) for 10 days to break seed dormancy. After that, we put them in a greenhouse compartment and kept them at 70% humidity and temperature between 15 and 25°C. In a preceding pilot experiment, we found that some species germinated faster than others. Therefore, we sowed the seeds of some of the species pairs (genera) 3 days later (see Online Appendix III) to ensure that seedlings would emerge more or less simultaneously.

### Experimental set-up

**Figure 1:**
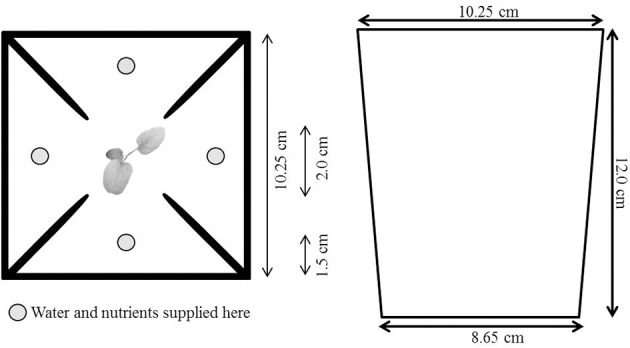
**Pot set-up**. We filled 1-L square pots with a 1:1 mixture of fine sand and fine vermiculite, and placed PVC barriers in the pots to create four pot quarters. The middle 2 × 2 cm was left open. We supplied plants with a 40ml nutrient solution three times a week with four syringes in the depicted spots (160 ml in total). Plants in the heterogeneous treatment received a high-strength nutrient solution (40 ml 1/2-strength Hoagland solution) in one quarter and a low-strength nutrient solution (40 ml 1/64-strength Hoagland solution) in the other three pot quarters. Plants in the homogeneous treatment received the same total amount of nutrients as in the other treatment but as equal intermediate-strength nutrient solutions (40 ml of ~1/8-strength Hoagland solution) in all four pot quarters.

We filled a total of 358 square 1-L pots with a 1:1 mixture of sand and fine vermiculite. Our experiment lasted for 5 weeks. This timespan enabled us to study early foraging responses as they may be particularly critical for the establishment of plants in nature and also allowed us to harvest the plants before the soil volume was limiting to the extent that we could no longer see foraging responses. To reduce the chances that roots would spill over from one quarter into the others due to limited root space and to prevent minimize nutrient leakage between pot quarters, we placed PVC barriers in the pots to divide them into four quarters (barriers stuck out above the soil, and were pushed all the way down). The central 2 × 2 cm of each pot was left barrier free to allow plants to grow roots in any direction (see Figure 1).

After the seedlings had been in the greenhouse for 2 weeks, we transplanted, if available, 16 plants per species into the center of the pots. Because some species germinated poorly, we had <16 plants for *Arctium tomentosum* (8 plants), *Centaurea scabiosa* (6), *Cirsium palustre* (15), *Linaria repens* (12), *Trifolium medium* (1), and *Veronica hederifolia* (9) (see Online Appendix III). The relatively low number of replicates for some of our species could have been problematic, if the objective would have been to get accurate values for each species in our experiment. However, we aimed to get representative values for the invasive and non-invasive species as a group and not as individual species. van Kleunen et al. (2014a) recently showed, using simulations, that the statistical power for detecting differences between groups of species increases with an increasing number of species used, even if the number of replicates per species becomes very low. Pots were placed 7 cm apart on a greenhouse bench, and their positions were fully randomized. At the start of the experiment, we counted the number of true leaves (i.e., excluding the cotyledons), and measured the length and width of the largest leaf on each plant.

Half of the pots of each species were assigned to a heterogeneous nutrient treatment; the other half was assigned to a homogeneous nutrient treatment. When we had an odd number of plants for a species, we maximized the number of replicates for the root-foraging measurements by allocating one plant more to the heterogeneous treatment than to the homogeneous treatment. Plants in the heterogeneous treatment received a high-strength nutrient solution (40 ml of a 1/2-strength Hoagland solution in one pot quarter and a low-strength nutrient solution (40 ml of a 1/64-strength Hoagland solution) in the other three pot quarters. Plants in the homogeneous treatment received the same total amount of nutrients as in the other treatment but as equal intermediate-strength nutrient solutions (40 ml of a c. 1/8-strength Hoagland solution) in all four pot quarters. We watered and fertilized all plants three times per week. Nutrients were supplied 1.5 cm from the pot border through four syringes, which simultaneously dripped the solution into the pot quarters at a rate of 25 ml/min. We tested whether we created a real nutrient gradient in the pots by taking soil samples from pots without plants in the first, third, and the last week of the experiment. We then analyzed the N content of these soil samples (see Online Appendix IV for more details on this procedure). The fertilization regime resulted in a 7.4- (first week of the experiment) to 29-fold (last week of the experiment) difference in N concentration between the high-nutrient and low-nutrient pot quarters in the heterogeneous treatment (Online Appendix IV).

### Measurements

Five weeks after the start of the experiment, species were harvested per congeneric species pair (see Online Appendix III for dates). We started with the pairs that had the largest plants. We cut the aboveground biomass at soil level, dried it for at least 72 h at 80°C, and weighed it. We divided the soil of each pot into four parts according to the pot quarters, and washed the roots from the soil. Thick tap roots and large storage roots (lignified roots, thicker than 2 mm) were separated from the other roots, as they contribute relatively little to nutrient uptake. Moreover, although these thick roots were usually in the middle of the pots, they could strongly bias the foraging results if by chance they ended up in one of the pot quarters. We determined the root length and diameter of the roots of the plants from the heterogeneous treatment. In preparation, we stained and preserved the roots from these plants in a neutral-red solution with 0.01% HgCl_2_ until further analysis. We then determined the length and diameter of all non-storage roots from each pot quarter using a scanner and WinRhizo software (Regent Instruments Inc., Quebec, Canada). Then all roots, also the ones of the plants in the homogeneous treatment, were dried for at least 72 h at 80°C, and weighed. For plants in the heterogeneous treatment, we calculated the specific root length for roots in the high-nutrient pot quarter and the opposing low-nutrient pot quarter as the root length divided by the root biomass.

### Statistical analyses

We analyzed our data with linear mixed models, using the *lme* function (Pinheiro et al., 2010) in the statistical program R (R development core team, 2010). With these models, it is possible to account for the complex nested design of our multi-species experiment by including it in the models as a random structure. These models are also relatively robust when data are unbalanced. Furthermore, the lme function allowed us to correct for heteroscedasticity, caused by the large differences in variance among the species, by adding species variances as a weighting factor (Zuur et al., 2009).

#### Testing the adaptive value of root foraging

The average performance of species may increase with their average foraging response, and the performance of an individual plant of a species may increase with its foraging response. As we used multiple species, we could test the adaptive value of plastic foraging responses in the heterogeneous nutrient treatment simultaneously at the among-species level and at the within-species level. We teased apart the effects of the strength of within- and among-species foraging responses on biomass production using a random regression model (e.g., Lane et al., 2012) with within-species mean centering. To do this, we first calculated a foraging index for each individual plant in the heterogeneous nutrient treatment. For root length, root biomass and specific root length, we calculated the index as (the trait value in the high-nutrient patch - the trait value in the opposite low-nutrient patch)/(the trait value in the high-nutrient patch + the trait value in the opposite low-nutrient patch) (e.g., Wang et al., 2013). This way, a high value would indicate a stronger root-foraging response. Because we expected plants to produce thinner roots in the high-nutrient patch, we calculated the foraging index for root diameter as (root diameter in the low-nutrient patch - root diameter in the high-nutrient patch)/(root diameter in the low-nutrient patch + root diameter in the high-nutrient patch). Then we calculated the average of each foraging index per species for each trait, and the deviation of each foraging index of each individual plant from the average foraging index of the species it belongs to. We used linear mixed effects models in which ln(total biomass) of individual plants in the heterogeneous nutrient treatment was used as the response variable, and the average species foraging index (i.e., to test the among-species effect) and the deviations of the individual foraging indices (i.e., to test the within-species effect) as explanatory variables. We included the initial size of the plants as a covariable. To account for variation among species and variation in the within-species effect among species, we included the random effects of species identity and initially allowed the slope of the effects of individual foraging index deviations to vary among species. Because random slopes did not significantly improve model fit in the models with foraging indices of root length, root diameter, and specific root length as explanatory variables, we removed the random slopes from those models.

#### Testing for differences between invasive and non-invasive species

For the subset of plants in the heterogeneous nutrient treatment, we tested whether there was a difference in foraging response between invasive and non-invasive plant species. For these analyses, we used as response variables root biomass (biomass of belowground parts excluding large storage structures), root length, root diameter, and specific root length. To quantify the strength of the foraging response, we compared the data from the high-nutrient quarter and the opposite low-nutrient quarter within each pot (i.e., we had two data points per pot). For the whole data set, we tested whether there was a difference in performance between invasive and non-invasive species in response to nutrient heterogeneity. As measures of plant performance, we used total biomass, aboveground biomass, and belowground biomass (all four pot quarters combined). We think that biomass production is a good proxy of performance as size is frequently associated with competitive ability (Dostál, 2011) and seed production (Shipley and Dion, 1992) in herbaceous plants.

For the analyses of foraging responses, the fixed terms of our models included invasiveness of the species (invasive and non-invasive), nutrient patch (high- and low-nutrient patch), their interaction and a measure of initial size of the plants (the length × width of the largest leaf × the number of true leaves). For the analyses of performance traits, the fixed model part included invasiveness of the species, nutrient treatment (homogeneous and heterogeneous treatment), their interaction and initial size of the plants. The hierarchical design of our experiment was included in the models as a nested random term: family/genus/species/pot. In the analyses of performance traits, we had only one value per pot, and accordingly we did not specify “pot” in the random part of these models (i.e., the variance among pots corresponds to the residual variance).

We used likelihood-ratio tests, based on maximum-likelihood estimation, to test which fixed factors were significant (Zuur et al., 2009). We first tested significance of the two-way interaction by removing it from the model and comparing this model to the full model. We then tested significance of the main effects by removing each one in turn and comparing these models to the full additive model (i.e., the model without the two-way interaction). To achieve normality of the residuals, we ln-transformed the data of root length, root biomass, belowground biomass, specific root length, aboveground biomass, and total biomass. For all analyses in which we used belowground biomass, we excluded the data from three plants (two *T. pratense* and one *R. acris*) because of accidentally mixed up roots.

## Results

### The adaptive value of root-foraging in heterogeneous soils

**Figure 2:**
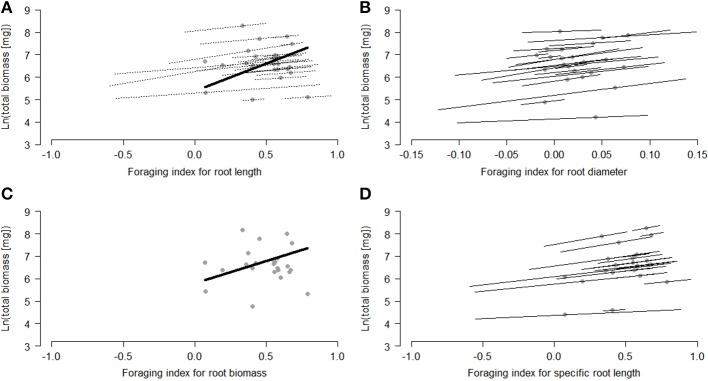
**Relationships between root-foraging indices and plant performance [ln (total plant biomass)] within and among the study species**. The modeled mean effects of each species are indicated by the dots. When the within-species relationships were significant or marginally significant, they are shown by the thin lines through each dot. When the among-species relationship was significant or marginally significant, it is shown by the thick line. Foraging responses were measured as **(A)** root length, **(B)** root diameter, **(C)** root biomass, and **(D)** specific root length, and performance as the ln-transformed total plant biomass. For calculation of the foraging index see the section ' Testing the adaptive value of root foraging Significant effects are presented as follows: within-species: *p* > 0.1 (no lines), 0.05 < *p* > 0.1 (dotted lines), *p* < 0.05 (solid lines); among-species: *p* > 0.1 (no line) and 0.1 < *p* > 0.05 (dotted line). The length of the line represents the within-species and among-species spread in the foraging index.

Within species, some strong individual root-foraging responses had positive effects on biomass production (Table 1, Figure 2). When the foraging response was measured in terms of root diameter and specific root length, these effects were significant. They were marginally significant when measured in terms of root length and non-significant when measured in terms of root biomass (Table 1, Figure 2). Among species, species with a stronger average foraging response in terms of root length and root biomass had a significantly higher biomass production than species with a weaker average foraging response (Table 1, Figure 2).

**Table 1:** **Effects of the strength of the within-species and between-species foraging responses (root length, root diameter, root biomass, specific root length) on the ln-transformed plant biomass**.

**Foraging trait**	**Among species**		**Within species**	
	**Estimate (± s.e.)**	***P*-value**	**Estimate (± s.e.)**	***P*-value**
Root length	**2.45 (1.04)**	**0.020**	0.62 (0.32)	0.054
Root diameter	5.64 (7.68)	0.464	**4.39 (1.75)**	**0.013**
Root biomass	**2.01 (0.87)**	**0.023**	−0.20 (0.34)	0.553
Specific root length	−0.93 (2.03)	0.646	**0.79 (0.25)**	**0.002**

*Presented are the fixed effects estimates of linear mixed effects models, the associated standard errors and p-values of the among-species and within-species effects. The scaled start size of the plants was used as a model covariable; species identity was used as a random factor. Significant results are in bold. Positive estimates indicate a positive effect of root foraging on performance*.

### Root foraging of invasive and non-invasive species in heterogeneous soils

**Figure 3:**
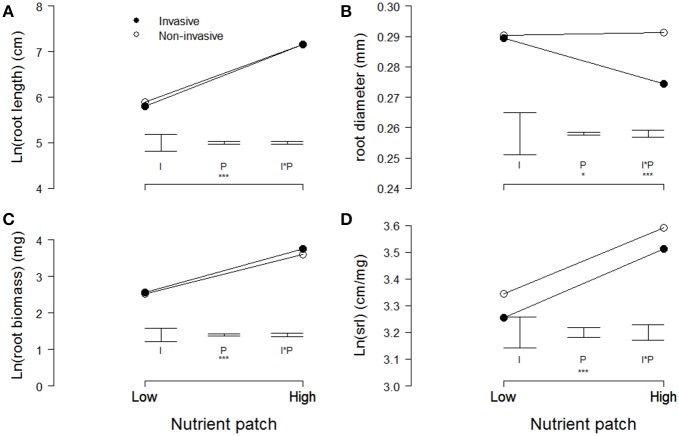
**Modeled means of (A) root length, (B) root diameter, (C) root biomass, and (D) the specific root length of invasive (filled symbols) and non-invasive (open symbols) plant species in the high-nutrient pot quarter and the opposing low-nutrient quarter**. Please note the cut y-axes. Error bars indicate the modeled standard errors for the effects of invasiveness (I), the nutrient patch (P), and the interaction between the two (I^*^P). Significant effects are presented as follows: 0.05 < *p* < 0.1 (.), 0.01 < *p* < 0.05 (^*^), 0.001 < *p* < 0.01 (^**^), *p* < 0.001 (^***^).

In the heterogeneous-nutrient treatment, plants produced significantly longer and thinner roots, and had more root biomass and a higher specific root length in the high-nutrient patches than in the low-nutrient patches (Table 2, Figure 3). Overall, average root length, root diameter, root biomass, and specific root length (i.e., root length/root biomass) did not differ significantly between invasive and non-invasive species (Table 2, Figure 3). However, the root-diameter foraging response was stronger in the invasive than in the non-invasive species (Figure 3B), as indicated by a significant invasiveness × nutrient patch interaction for root diameter (Table 2).

**Table 2:** **Effects of high- and low-nutrient patches of the heterogeneous treatment on the biomass and morphology of roots of invasive and non-invasive plant species**.

**Response variable**	**Initial size**	**Invasiveness (I)**	**Nutrient patch (P)**	I^*^P
Root length	**7.51 (0.006)**	0.02 (0.894)	**272.99 (0.000)**	1.10 (0.294)
Root diameter	1.15 (0.283)	0.42 (0.515)	**4.09 (0.043)**	**21.86 (0.000)**
Root biomass	0.78 (0.376)	0.07 (0.797)	**180.40 (0.000)**	1.22 (0.268)
Specific root length	**3.86 (0.049)**	0.54 (0.463)	**43.42 (0.000)**	0.03 (0.865)

*Presented are the results of likelihood-ratio tests [log-likelihood ratio and corresponding p-value (in parentheses)], which we used to test whether invasiveness of species, high- or low-nutrient patches and their interaction significantly contributed to the fit of linear mixed effects models. Significant results are in bold*.

### Performance of invasive and non-invasive species in homogeneous and heterogeneous soils

**Figure 4:**
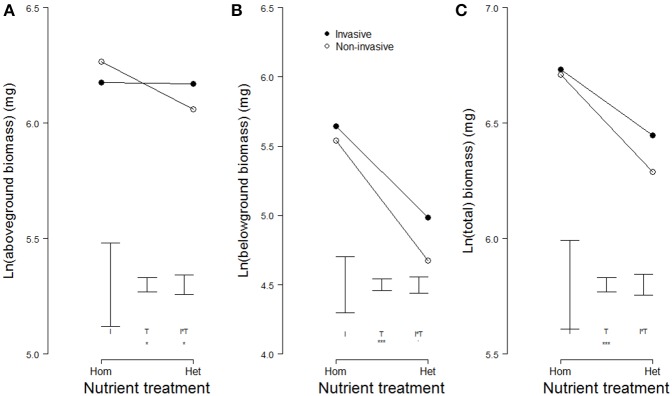
**Modeled means of (A) aboveground biomass, (B) belowground biomass, and (C) total biomass of invasive (filled symbols) and non-invasive (open symbols) plant species growing in pots with homogeneously or heterogeneously distributed soil nutrients**. Please note the cut y-axes. Error bars indicate the modeled standard errors for the effects of invasiveness (I), the nutrient treatment (T), and the interaction between the two (I^*^T). Significant effects are presented as follows: 0.05 < *p* < 0.1 (.), 0.01 < *p* < 0.05 (^*^), 0.001 < *p* < 0.01 (^**^), *p* < 0.001 (^***^).

Averages of aboveground, belowground, and total biomass did not differ significantly between invasive and non-invasive species (Table 3, Figure 4C), most likely because we *a priori* selected congeneric species of similar size. Overall, plants produced more biomass in the homogeneous than in the heterogeneous treatment (Table 3, Figures 4A–C). However, while the non-invasive species had reduced aboveground biomass in the heterogeneous treatment, the invasive species had not (significant two-way interaction in Table 3, Figure 4A).

**Table 3:** **Effects of nutrient heterogeneity on the performance (aboveground biomass, belowground biomass, and total plant biomass) of invasive and non-invasive plant species**.

**Response variable**	**Initial size**	**Invasiveness (I)**	**Nutrient treatment (T)**	I^*^T
Aboveground biomass	**23.65 (0.000)**	0.00 (0.975)	**4.08 (0.043)**	**5.33 (0.021)**
Belowground biomass	**4.92 (0.027)**	0.28 (0.599)	**91.36 (0.000)**	2.88 (0.090)
Total biomass	**19.27 (0.000)**	0.06 (0.811)	**35.28 (0.000)**	2.35(0.125)

*Presented are the results of likelihood-ratio tests [log-likelihood ratio and corresponding p-value (in parentheses)], which we used to test whether invasiveness of species, soil nutrient heterogeneity and their interaction significantly improved the model fit of linear mixed effects models. Significant results are in bold*.

## Discussion

Our results suggest that root-foraging responses are adaptive in heterogeneous nutrient environments, because we found significant positive effects of root foraging on biomass production of individual plants within species, and significant positive effects of foraging on biomass production of species (Table 1, Figure 2). We also found stronger root-diameter foraging responses in invasive compared to non-invasive plant species (Table 2, Figure 3B). In addition, although plants performed worse in the heterogeneous than in the homogeneous nutrient environment overall, invasive species were better able than non-invasive species of maintaining a relatively high aboveground biomass production in the heterogeneous nutrient treatment (Table 3, Figures 4A–C). Together, these findings suggest that root foraging is adaptive, and might aid species in becoming invasive in natural areas elsewhere.

Although it is frequently implicitly assumed that phenotypic plasticity of a trait in response to an environmental cue is adaptive, there are still surprisingly few plant traits and environmental factors for which this has been empirically tested (van Kleunen and Fischer, 2005). Well-studied examples are shade-avoidance plasticity, such as stem elongation (Dudley and Schmitt, 1996; van Kleunen and Fischer, 2001), and induced resistance against herbivores (Agrawal, 1998; Agrawal et al., 2002; Crispo, 2007). Despite the large number of studies on plastic root foraging (e.g., Robinson et al., 1999; Kembel et al., 2008), we found only one other experimental study in which its adaptive value was explicitly tested. Wang et al. (2013) found that, in a high-contrast nutrient environment, genotypes of *Potentilla reptans* with a stronger foraging response, measured in terms of root biomass allocation, had a higher total biomass than genotypes with a weaker response. Here, we showed that this holds across a much larger number of herbaceous species.

The strength of the foraging response and its effects on performance can vary with the strength of the nutrient contrast (Wijesinghe and Hutchings, 1999; Wang et al., 2013), and the persistence of nutrient patches in time (e.g., Mommer et al., 2011) and space (e.g., Fransen et al., 1999). If these patch dynamics in nature are faster than the root-foraging responses of the plants, the latter could potentially result in maladaptation (Stuefer, 1996; Dewitt, 1998). However, as long as the foraging response results in at least a temporarily higher nutrient acquisition, it could provide a competitive advantage, and result in a longer term benefit. Another strategy that could allow alien plants to take advantage of resource pulses is to rapidly germinate after a resource pulse. Indeed it has been reported that successful alien species frequently germinate faster than less successful alien species (van Kleunen and Johnson, 2007; Schlaepfer et al., 2010). Recently, Wilsey et al. (2015) showed that invasive species can take advantage of such priority effects. Future studies should address whether invasive species are in general better in taking advantage of resource pulses in dynamic environments, and which strategies they use.

We found that the invasive species showed a more plastic root-diameter foraging response than the non-invasive species (Table 2, Figure 3B). Similarly, Keser et al. (2014) recently found that invasive clonal plants showed stronger root-biomass foraging responses than non-invasive clonal plants. These results are consistent with the idea that invasive species should have stronger phenotypic plasticity than non-invasive species (Baker, 1965; Richards et al., 2006; Davidson et al., 2011; Palacio-López and Gianoli, 2011). Even though invasive species are expected to be more plastic, many empirical tests did not find support for this (Schlaepfer et al., 2010; Palacio-López and Gianoli, 2011; van Kleunen et al., 2011). Several factors may contribute to the lack of support for this hypothesis in other studies. First, and in contrast to our study, most of the studies did not test whether plasticity in the trait studied was adaptive. Second, many studies did not use genetically identical plant material in the different treatments, and thus may have confounded phenotypic plasticity with genetic differences, if there was genotype-by-environment covariation (Schmid, 1992). We avoided this problem by looking at phenotypic plasticity at the within-plant level. Third, not all studies compared invasive alien species to less invasive alien species, but focused on differences between invasive alien and native species (Davidson et al., 2011; Palacio-López and Gianoli, 2011). Although such comparisons might provide insights into why invasive species can displace certain native species, they do not test why some alien species become invasive and others do not (van Kleunen et al., 2010a; Burns et al., 2013).

One of the assumptions of the benefit of adaptive phenotypic plasticity for invasiveness is that plastic species could express optimal phenotypes under different growing conditions, and are therefore pre-adapted to become invasive (Richards et al., 2006). We therefore expected that the invasive species would be better capable than the non-invasive species of taking advantage of the high nutrient patches, and thus of maintaining a relatively high performance in the heterogeneous nutrient environment. Indeed, we found this for aboveground biomass, and this suggests that plasticity may aid invasive species to grow better under less favorable heterogeneous growing conditions. However, we did not find significant advantages in terms of belowground and total biomass. Possibly, the experiment did not last long enough for such advantages to become apparent. Furthermore, it has been reported that root foraging may increase competitive potential (Robinson et al., 1999). Therefore, future studies should test whether foraging under competitive conditions increases the performance of invasive species over non-invasive species.

Many other plant characteristics that have been reported to be positively correlated with species invasiveness are also related to nutrient responses. Dostál et al. (2013) found that Central European plant species from more productive habitats and species with a wider habitat-productivity niche in their native range have higher success as alien plant species elsewhere in the world. Dawson et al. (2012a) found that invasive species, just like common native species, can capitalize more strongly on extra nutrients than non-invasive alien species, and rare native species, do. Similarly, Dawson et al. (2012b) found that alien plant species with a wider global distribution are better able to capitalize on increased resource availability. Furthermore, Funk and Vitousek (2007) found that in nutrient-poor environments, the nutrient-uptake efficiency of invasive species was higher than that of native species. The ability to effectively exploit soil nutrients may therefore be one of the important factors determining invasiveness of plant species.

Natural areas can be competitive environments, and this may hamper colonization by new species. Strong root foraging could aid colonizing species by allowing them to exploit the available nutrients, and this has been reported to increase the competitive potential of plant species (Robinson et al., 1999). However, Mommer et al. (2012) found that, although a competitively strong species benefited from foraging under competition, a competitively weaker species had a disadvantage because it placed its roots in the empty soil patches instead of in the nutrient-rich patches. Interestingly, we found that on average natural-area invaders had a stronger foraging response than non-invaders. Invasive plant species have been hypothesized to have a stronger competitive ability than non-invasive species (Baker, 1974), although few studies have tested this explicitly (Dawson et al., 2012a). If invasive species are indeed competitively stronger and have a stronger foraging response, this could mean additive benefits for the invasive species in comparison to non-invasive species.

It has been suggested that the importance of certain plant traits for plant invasiveness may change with the stage of the invasion (Pyšek, 1997; Dietz and Edwards, 2006; Theoharides and Dukes, 2007; Dawson et al., 2009; Pyšek et al., 2009). Here, we focused on a transition that has received little research attention: the transition from being naturalized to invading natural areas. We think that this transition deserves special attention, because species that can invade natural areas are likely to become problematic invaders. Our experiment indicates that the potential for nutrient foraging may contribute to species invasiveness in this transition.

### Conflict of interest statement

The Guest Associate Editor Judy Simon declares that, despite being affiliated with the same institute as the authors Lidewij Hester Keser, Wayne Dawson and Mark Van Kleunen, the review process was handled objectively. The authors declare that the research was conducted in the absence of any commercial or financial relationships that could be construed as a potential conflict of interest.

## References

[B1] Agrawal A. A. (1998). Induced responses to herbivory and increased plant performance. Science 279, 1201–1202. 10.1126/science.279.5354.1201 9469809

[B2] Agrawal A. A. Conner J. K. Johnson M. T. Wallsgrove R. (2002). Ecological genetics of an induced plant defense against herbivores: additive genetic variance and costs of phenotypic plasticity. Evolution 56, 2206–2213. 10.1554/0014-3820(2002)056[2206:EGOAIP]2.0.CO;2 12487351

[B3] Baker H. G. (1965). Characteristics and modes of origin of weeds, in The Genetics of Colonizing Species, eds Baker H. G. Stebbins G. L. (New York; London: Academic Press), 147–168.

[B4] Baker H. G. (1974). The evolution of weeds. Annu. Rev. Ecol. Syst. 5, 1–24. 10.1146/annurev.es.05.110174.0002459789628

[B5] Bradshaw A. (1965). Evolutionary significance of phenotypic plasticity in plants. Adv. Genet. 13, 115–155 10.1016/S0065-2660(08)60048-6

[B6] Burns J. H. Pardini E. A. Schutzenhofer M. R. Chung Y. A. Seidler K. J. Knight T. M. (2013). Greater sexual reproduction contributes to differences in demography of invasive plants and their noninvasive relatives. Ecology 94, 995–1004. 10.1890/12-1310.1 23858640

[B7] Crispo E. (2007). The Baldwin effect and genetic assimilation: revisiting two mechanisms of evolutionary change mediated by phenotypic plasticity. Evolution 61, 2469–2479. 10.1111/j.1558-5646.2007.00203.x 17714500

[B8] Davidson A. M. Jennions M. Nicotra A. B. (2011). Do invasive species show higher phenotypic plasticity than native species and, if so, is it adaptive? A meta-analysis. Ecol. Lett. 14, 419–431. 10.1111/j.1461-0248.2011.01596.x 21314880

[B9] Davis M. A. Grime J. P. Thompson K. (2000). Fluctuating resources in plant communities: a general theory of invasibility. J. Ecol. 88, 528–534. 10.1046/j.1365-2745.2000.00473.x16026814

[B10] Dawson W. Burslem D. F. R. P. Hulme P. E. (2009). Factors explaining alien plant invasion success in a tropical ecosystem differ at each stage of invasion. J. Ecol. 97, 657–665 10.1111/j.1365-2745.2009.01519.x

[B11] Dawson W. Fischer M. van Kleunen M. (2011). The maximum relative growth rate of common UK plant species is positively associated with their global invasiveness. Glob. Ecol. Biogeogr. 20, 299–306 10.1111/j.1466-8238.2010.00599.x

[B12] Dawson W. Fischer M. van Kleunen M. (2012a). Common and rare plant species respond differently to fertilization and competition, whether they are alien or native. Ecol. Lett. 15, 873–880. 10.1111/j.1461-0248.2012.01811.x 22676338

[B13] Dawson W. van Kleunen M. Rohr R. Fischer M. (2012b). Alien plant species with a wider global distribution are better able to capitalize on increased resource availability. New Phytol. 194, 859–867. 10.1111/j.1469-8137.2012.04104.x 22409575

[B14] de Kroon H. Visser E. J. Huber H. Mommer L. Hutchings M. J. (2009). A modular concept of plant foraging behaviour: the interplay between local responses and systemic control. Plant Cell Environ. 32, 704–712. 10.1111/j.1365-3040.2009.01936.x 19183298

[B15] Dewitt T. J. (1998). Costs and limits of phenotypic plasticity: tests with morphology and life history in a freshwater snail. J. Evol. Biol. 11, 465–480 10.1007/s000360050100

[B16] Dietz H. Edwards P. J. (2006). Recognition that causal processes change during plant invasion helps explain conflicts in evidence. Ecology 87, 1359–1367. 10.1890/0012-9658(2006)87[1359:RTCPCD]2.0.CO;2 16869409

[B17] Dostál P. (2011). Plant competitive interactions and invasiveness: searching for the effects of phylogenetic relatedness and origin on competition intensity. Am. Nat. 177, 655–67. 10.1086/659060 21508611

[B18] Dostál P. Dawson W. van Kleunen M. Keser L. H. Fischer M. (2013). Central European plant species from more productive habitats and with wider productivity niches are more invasive at a global scale. Glob. Ecol. Biogeogr. 22, 64–72 10.1111/j.1466-8238.2011.00754.x

[B19] Dudley S. Schmitt J. (1996). Testing the adaptive plasticity hypothesis: density-dependent selection on manipulated stem length in Impatiens capensis. Am. Nat. 147, 445–465 10.1086/285860

[B20] Fransen B. Blijjenberg J. de Kroon H. (1999). Root morphological and physiological plasticity of perennial grass species and the exploitation of spatial and temporal heterogeneous nutrient patches. Plant Soil 211, 179–189 10.1023/A:1004684701993

[B21] Funk J. L. Vitousek P. M. (2007). Resource-use efficiency and plant invasion in low-resource systems. Nature 446, 1079–1081. 10.1038/nature05719 17460672

[B22] Grotkopp E. Rejmanek M. (2007). High seedling relative growth rate and specific leaf area are traits of invasive species: phylogenetically independent contrasts of woody angiosperms. Am. J. Bot. 94, 526–532. 10.3732/ajb.94.4.526 21636422

[B23] Hodge A. (2004). The plastic plant: root responses to heterogeneous supplies of nutrients. New Phytol. 162, 9–24 10.1111/j.1469-8137.2004.01015.x

[B24] Hulme P. (2008). Phenotypic plasticity and plant invasions: is it all Jack? Funct. Ecol. 22, 3–7 10.1111/j.1365-2435.2007.01369.x

[B25] Kembel S. W. Cahill J. F. Jr. (2005). Plant phenotypic plasticity belowground: a phylogenetic perspective on root foraging trade-offs. Am. Nat. 166, 216–230. 10.1086/431287 16032575

[B26] Kembel S. W. de Kroon H. Cahill J. F. Jr Mommer L. (2008). Improving the scale and precision of hypotheses to explain root foraging ability. Ann. Bot. 101, 1295–1301. 10.1093/aob/mcn044 18424813 PMC2710254

[B27] Keser L. H. Dawson W. Song Y. Yu F.-H. Fischer M. Dong M.. (2014). Invasive clonal plant species have a greater root-foraging plasticity than non-invasive ones. Oecologia 174, 1055–1064. 10.1007/s00442-013-2829-y 24352844

[B28] Lane J. E. Kruuk L. E. Charmantier A. Murie J. O. Dobson F. S. (2012). Delayed phenology and reduced fitness associated with climate change in a wild hibernator. Nature 489, 554–557. 10.1038/nature11335 22878721

[B29] Mommer L. van Ruijven J. Jansen C. van de Steeg H. M. de Kroon H. (2012). Interactive effects of nutrient heterogeneity and competition: implications for root foraging theory? Funct. Ecol. 26, 66–73 10.1111/j.1365-2435.2011.01916.x

[B30] Mommer L. Visser E. J. W. Ruijven J. Caluwe H. Pierik R. de Kroon H. (2011). Contrasting root behaviour in two grass species: a test of functionality in dynamic heterogeneous conditions. Plant Soil 344, 347–360 10.1007/s11104-011-0752-8

[B31] Palacio-López K. Gianoli E. (2011). Invasive plants do not display greater phenotypic plasticity than their native or non-invasive counterparts: a meta-analysis. Oikos 120, 1393–1401 10.1111/j.1600-0706.2010.19114.x

[B32] Parepa M. Fischer M. Bossdorf O. (2013). Environmental variability promotes plant invasion. Nat. Commun. 4:1604. 10.1038/ncomms2632 23511469

[B33] Pinheiro J. Bates D. DebRoy S. Sarkar D. R development core team. (2010). nlme: Linear and Nonlinear Mixed Effects Models. Vienna: R Foundation for Statistical Computing.

[B34] Pyšek P. (1997). Clonality and plant invasions: can a trait make a difference, in The Ecology and Evolution of Clonal Plants, eds de Kroon H. Van Groenendael J. M. (Leiden: Backhuys Publishers), 405–427.

[B35] Pyšek P. Jarošík V. Pergl J. Randall R. Chytrý M. Kühn I. (2009). The global invasion success of Central European plants is related to distribution characteristics in their native range and species traits. Divers. Distrib. 15, 891–903 10.1111/j.1472-4642.2009.00602.x

[B36] Pyšek P. Richardson D. M. (2007). Traits associated with invasiveness in alien plants: where do we stand?, in Biological Invasions, ed Nentwig W. (Berlin; Heidelberg: Springer Verlag), 97–126.

[B37] R development core team. (2010). R: A Language and Environment for Statistical Computing, Reference Index Version 2.12.0. Vienna: R Foundation for Statistical Computing.

[B38] Richards C. L. Bossdorf O. Muth N. Z. Gurevitch J. Pigliucci M. (2006). Jack of all trades, master of some? On the role of phenotypic plasticity in plant invasions. Ecol. Lett. 9, 981–993. 10.1111/j.1461-0248.2006.00950.x 16913942

[B39] Richardson D. M. Pyšek P. Rejmánek M. Barbour M. G. Panetta F. D. West C. J. (2000). Naturalization and invasion of alien plants: concepts and definitions. Divers. Distrib. 6, 93–107 10.1046/j.1472-4642.2000.00083.x

[B40] Robinson D. Hodge A. Griffiths B. S. Fitter A. H. (1999). Plant root proliferation in nitrogen-rich patches confers competitive advantage. Proc. R. Soc. London. Ser. B Biol. Sci. 266, 431–435 10.1098/rspb.1999.0656

[B41] Rothmaler W. Jaeger E. J. Werner K. (2005). Excursionsflora von Deutschland, Band 4, 10th Edn. Muenchen: Elsevier GmbH.

[B42] Schlaepfer D. R. Glättli M. Fischer M. van Kleunen M. (2010). A multi-species experiment in their native range indicates pre-adaptation of invasive alien plant species. New Phytol. 185, 1087–1099. 10.1111/j.1469-8137.2009.03114.x 19968796

[B43] Schmid B. (1992). Phenotypic variation in plants. Evol. Trends Plants 6, 45–60.

[B44] Shipley B. Dion J. (1992). The allometry of seed production in herbaceous angiosperms. Am. Nat. 139, 467–483 10.1086/285339

[B45] Stuefer J. F. (1996). Potential and limitations of current concepts regarding the response of clonal plants to environmental heterogeneity. Vegetatio 127, 55–70 10.1007/BF00054847

[B46] Theoharides K. A. Dukes J. S. (2007). Plant invasion across space and time: factors affecting nonindigenous species success during four stages of invasion. New Phytol. 176, 256–273. 10.1111/j.1469-8137.2007.02207.x 17822399

[B47] van Kleunen M. Dawson W. Bossdorf O. Fischer M. (2014a). The more the merrier: multi-species experiments in ecology. Basic Appl. Ecol. 15, 1–9 10.1016/j.baae.2013.10.006

[B48] van Kleunen M. Dawson W. Maurel N. (2014b). Characteristics of successful alien plants. Mol. Ecol. 10.1111/mec.13013 [Epub ahead of print]. 25421056

[B49] van Kleunen M. Dawson W. Schlaepfer D. R. Jeschke J. M. Fischer M. (2010a). Are invaders different? A conceptual framework of comparative approaches for assessing determinants of invasiveness. Ecol. Lett. 13, 947–958. 10.1111/j.1461-0248.2010.01503.x 20576028

[B50] van Kleunen M. Fischer M. (2001). Adaptive evolution of plastic foraging responses in a clonal plant. Ecology 82, 3309 10.2307/2680154

[B51] van Kleunen M. Fischer M. (2005). Constraints on the evolution of adaptive phenotypic plasticity in plants. New Phytol. 166, 49–60. 10.1111/j.1469-8137.2004.01296.x 15760350

[B52] van Kleunen M. Johnson S. D. (2007). South African Iridaceae with rapid and profuse seedling emergence are more likely to become naturalized in other regions. J. Ecol. 95, 674–681 10.1111/j.1365-2745.2007.01250.x

[B53] van Kleunen M. Schlaepfer D. R. Glaettli M. Fischer M. (2011). Preadapted for invasiveness: do species traits or their plastic response to shading differ between invasive and non-invasive plant species in their native range? J. Biogeogr. 38, 1294–1304 10.1111/j.1365-2699.2011.02495.x

[B54] van Kleunen M. Weber E. Fischer M. (2010b). A meta-analysis of trait differences between invasive and non-invasive plant species. Ecol. Lett. 13, 235–245. 10.1111/j.1461-0248.2009.01418.x 20002494

[B55] Wang Z. van Kleunen M. During H. Werger M. (2013). Root foraging increases performance of the clonal plant *Potentilla reptans* in heterogeneous nutrient environments. PLoS ONE 8:e58602. 10.1371/journal.pone.0058602 23472211 PMC3589344

[B56] Wijesinghe D. K. Hutchings M. J. (1999). The effects of environmental heterogeneity on the performance of *Glechoma hederacea*: the interactions between patch contrast and patch scale. J. Ecol. 87, 860–872 10.1046/j.1365-2745.1999.00395.x

[B57] Williamson M. Fitter A. (1996). The varying success of invaders. Ecology 77, 1661–1666 10.2307/2265769

[B58] Wilsey B. J. Barber K. Martin L. M. (2015). Exotic grassland species have stronger priority effects than natives regardless of whether they are cultivated or wild genotypes. New Phytol. 205, 928–937. 10.1111/nph.13028 25252271

[B59] Zuur A. F. Ieno E. N. Walker N. J. Saveliev A. A. Smith G. M. (2009). Mixed Effects Models and Extensions in Ecology with R. New York, NY: Springer 10.1007/978-0-387-87458-6

