# Supplementary material for: Herbaceous plant species invading natural areas tend to have stronger adaptive root foraging than other naturalized species
Source: Front Plant Sci. 2015 Apr 27;6:273. doi: 10.3389/fpls.2015.00273 (PMC4410514; doi:10.3389/fpls.2015.00273)
Supplement: Supplementary file 1 [file Table1.DOCX]

Online appendix I: Species details

The European plant species we used and the number of USA states in which the species is considered a natural-area invader, the number of USA states and Canadian provinces and territories in which the species is present, the average height, and growth form of these species. Invasive species are in bold.

| **Plant family** | **Species name** | **Natural-area invader status*** | **Number of states in which present**† | **Average height (cm)**‡ | **Growth form**§ |
| --- | --- | --- | --- | --- | --- |
| Asteraceae | *Arctium tomentosum* | - | 32 | 90 | B |
|  | ***Arctium minus*** | **16** | **47** | **90** | **B** |
|  | *Centaurea scabiosa* | - | 14 | 85 | P |
|  | ***Centaurea jacea*** | **4** | **26** | **85** | **P** |
|  | *Cirsium palustre* | - | 5 | 100 | H |
|  | ***Cirsium vulgare*** | **24** | **50** | **105** | **B** |
| Boraginaceae | *Myosotis arvensis* | - | 39 | 55 | B |
|  | ***Myosotis scorpioides*** | **4** | **39** | **55** | **P** |
| Caryophyllaceae | *Cerastium glomeratum* | - | 47 | 25 | A |
|  | ***Cerastium fontanum*** | **4** | **50** | **30** | **P** |
| Fabaceae | *Melilotus altissimus* | - | 8 | 90 | B |
|  | ***Melilotus officinalis*** | **26** | **51** | **65** | **B** |
|  | *Trifolium pratense* | - | 51 | 45 | P |
|  | ***Trifolium medium*** | **6** | **6** | **30** | **P** |
| Plantaginaceae | *Linaria repens* | - | 9 | 40 | P |
|  | ***Linaria vulgaris*** | **12** | **49** | **50** | **P** |
|  | *Plantago media* | - | 11 | 30 | P |
|  | ***Plantago major*** | **8** | **64** | **25** | **P** |
|  | *Veronica agrestis* | - | 27 | 20 | A |
|  | ***Veronica hederifolia*** | **5** | **27** | **25** | **A** |
| Polygonaceae | *Rumex acetosa* | - | 18 | 65 | P |
|  | ***Rumex crispus*** | **15** | **51** | **90** | **P** |
| Ranunculaceae | *Ranunculus arvensis* | - | 23 | 40 | A |
|  | ***Ranunculus acris*** | **4** | **40** | **75** | **P** |

* http://www.invasiveplantatlas.org, visited April 2010. Depicted is the number of US states in which the species is considered to be a natural-area invader.

† http://plants.usda.gov, visited June 2009. Depicted is the number of USA states and Canadian provinces and territories in which a species is present.

‡ Average height was calculated by averaging the minimum and the maximum height of the species as listed in the Exkursionsflora von Deutschland (Rothmaler et al. 2005).

§ Growth form of the species: A: annual, B: biennial, H: pluriennial-hapaxanthic, P: perennial
