# Supplementary material for: Herbaceous plant species invading natural areas tend to have stronger adaptive root foraging than other naturalized species
Source: Front Plant Sci. 2015 Apr 27;6:273. doi: 10.3389/fpls.2015.00273 (PMC4410514; doi:10.3389/fpls.2015.00273)
Supplement: Supplementary file 2 [file Table2.DOCX]

Online appendix II: Seed suppliers

| Species name | Wildstaudengärtnerei Eschenbach | Artha samen | Emorsgate seeds | UfA samen | Botanical garden Yves Rocher | Botanical garden Konstanz | Botanical garden Hohenheim | Botanical garden Regensburg | Botanical garden Turku | Botanical garden Kiel | Botanical garden Nantes | Botanical garden Krefeld | Botanical garden Göttingen |
| --- | --- | --- | --- | --- | --- | --- | --- | --- | --- | --- | --- | --- | --- |
| *Arctium tomentosum* |  |  |  |  |  | X |  |  |  |  |  |  |  |
| *Arctium minus* |  |  | X |  |  | X |  |  |  |  |  |  |  |
| *Centaurea scabiosa* | X | X | X | X |  |  |  |  |  |  |  |  |  |
| *Centaurea jacea* | X |  |  |  |  | X |  |  |  |  |  |  |  |
| *Cerastium fontanum* |  |  | X |  |  |  |  |  | X |  |  |  |  |
| *Cerastium glomeratum* |  |  |  |  |  | X |  |  |  |  |  |  |  |
| *Cirsium palustre* |  |  |  |  |  | X |  |  |  |  |  |  |  |
| *Cirsium vulgare* | X | X |  |  |  | X |  |  |  |  |  |  |  |
| *Hypericum perforatum* | X | X | X | X |  | X |  |  |  |  |  |  |  |
| *Linaria repens* |  |  |  |  |  | X |  |  |  |  |  |  |  |
| *Linaria vulgaris* | X | X | X | X |  | X |  |  |  |  |  |  |  |
| *Melilotus officinalis* | X |  |  |  |  |  | X |  |  |  |  |  |  |
| *Melilotus altissimus* |  |  |  |  |  | X |  |  |  |  |  |  |  |
| *Myosotis scorpioides* | X |  |  | X |  |  |  |  |  |  |  |  |  |
| *Myosotis arvensis* | X | X | X |  |  | X |  |  |  |  |  |  |  |
| *Persicaria maculosa* |  |  |  |  |  | X |  |  |  | X | X |  |  |
| *Plantago major* | X | X |  | X | X |  |  |  |  |  |  |  |  |
| *Plantago media* | X | X |  | X | X |  |  |  |  |  |  |  |  |
| *Ranunculus arvensis* | X |  | X | X |  | X |  |  |  |  |  |  |  |
| *Ranunculus acris* |  | X | X |  |  | X |  |  |  |  |  |  |  |
| *Rumex crispus* |  |  |  |  |  | X |  |  |  |  |  |  |  |
| *Rumex acetosa* | X | X | X |  | X |  | X |  |  |  |  |  |  |
| *Tragopogon pratensis* | X |  | X |  |  |  |  |  |  |  |  |  |  |
| *Tragopogon dubius* |  | X |  |  |  | X |  |  |  |  |  |  |  |
| *Trifolium medium* |  |  | X |  |  |  |  | X |  |  |  | X |  |
| *Trifolium pratense* |  | X | X | X | X |  |  | X |  |  |  |  |  |
| *Verbascum lychnitis* | X | X |  | X |  |  |  |  |  |  |  |  |  |
| *Verbascum thapsus* | X |  | X | X |  | X |  |  |  |  |  |  |  |
| *Veronica agrestis* |  |  |  |  |  | X |  |  |  |  |  |  |  |
| *Veronica hederifolia* |  |  |  |  |  | X |  |  |  |  |  |  |  |
