# Supplementary material for: Herbaceous plant species invading natural areas tend to have stronger adaptive root foraging than other naturalized species
Source: Front Plant Sci. 2015 Apr 27;6:273. doi: 10.3389/fpls.2015.00273 (PMC4410514; doi:10.3389/fpls.2015.00273)
Supplement: Supplementary file 3 [file Table3.DOCX]

Online appendix III: Plants used in the experiments (invasive species are in grey rows)

For most species, we had eight plants per treatment (i.e. 16 plants in total), but for some species we had lower numbers due to poor seed germination. Listed below are the used number of seedlings per species per treatment, the sowing date and the date the species pair was harvested. Some species pairs were sown earlier because of their slow germination rates. This difference in sowing dates allowed us to use similarly aged seedlings across all species in the experiment.

| **Species** | **Number of plants homogeneous** | **Number of plants heterogeneous** | **Sowing date** | **Harvest date** |
| --- | --- | --- | --- | --- |
| *Arctium tomentosum* | 4 | 4 | June 14 | August 23 |
| *Arctium minus* | 8 | 8 | June 14 | August 23 |
| *Centaurea scabiosa* | 3 | 3 | June 14 | September 5 |
| *Centaurea jacea* | 8 | 8 | June 14 | September 5 |
| *Cerastium glomeratum* | 8 | 8 | June 14 | September 1 |
| *Cerastium fontanum* | 8 | 8 | June 14 | September 1 |
| *Cirsium palustre* | 7 | 8 | June 14 | August 30 |
| *Cirsium vulgare* | 8 | 8 | June 14 | August 30 |
| *Linaria repens* | 6 | 6 | June 17 | August 31 |
| *Linaria vulgaris* | 8 | 8 | June 17 | August 31 |
| *Melilotus altissimus* | 8 | 8 | June 14 | August 23 |
| *Melilotus officinalis* | 8 | 8 | June 14 | August 23 |
| *Myosotis arvensis* | 8 | 8 | June 17 | August 30 |
| *Myosotis scorpioides* | 8 | 8 | June 17 | August 30 |
| *Plantago media* | 8 | 8 | June 17 | August 29 |
| *Plantago major* | 8 | 8 | June 17 | August 29 |
| *Ranunculus arvensis* | 8 | 8 | June 17 | August 25 |
| *Ranunculus acris* | 8 | 8 | June 17 | August 25 |
| *Rumex acetosa* | 8 | 8 | June 14 | August 23 |
| *Rumex crispus* | 8 | 8 | June 14 | August 23 |
| *Trifolium pratense* | 8 | 8 | June 14 | August 25 |
| *Trifolium medium* | 1 | 1 | June 14 | August 25 |
| *Veronica agrestis* | 8 | 8 | June 14 | September 2 |
| *Veronica hederifolia* | 4 | 5 | June 14 | September 2 |
