# Supplementary material for: Herbaceous plant species invading natural areas tend to have stronger adaptive root foraging than other naturalized species
Source: Front Plant Sci. 2015 Apr 27;6:273. doi: 10.3389/fpls.2015.00273 (PMC4410514; doi:10.3389/fpls.2015.00273)
Supplement: Supplementary file 4 [file Table4.DOCX]

Online appendix IV: Soil-nutrient contrasts

To test whether the heterogeneous fertilizer application resulted in a clear nutrient contrast between pot quarters, we also applied the heterogeneous fertilization treatment to 10 pots in which no plants were present. In the first, third and fifth week of the experiment, we took soil samples from four of these pots and analyzed the nitrogen concentrations in the soil solution. Two pots were sampled just before fertilization and another two were sampled just after fertilization. In week one, we only sampled after fertilization. Nine samples per pot were taken as depicted in Fig. 1 and frozen until further analysis.

We added 10 g soil to 25 ml of a 2M KCL solution, and shook this suspension for 15 minutes on an orbital shaker (speed dial 120). Then we left it to settle for 1 minute, poured the suspension fluid through a #1 Whatman paper, and froze the filtrate until nitrogen-content analysis. The nitrogen content (NO­_2_^-^ and NO_3_^-^­­) of the filtrate was analyzed using a segmented flow auto-analyzer (Technicon® AutoAnalyzer II, Technicon®). Per sample, 4 ml was analyzed.

Values within the quarters were averaged before presentation.

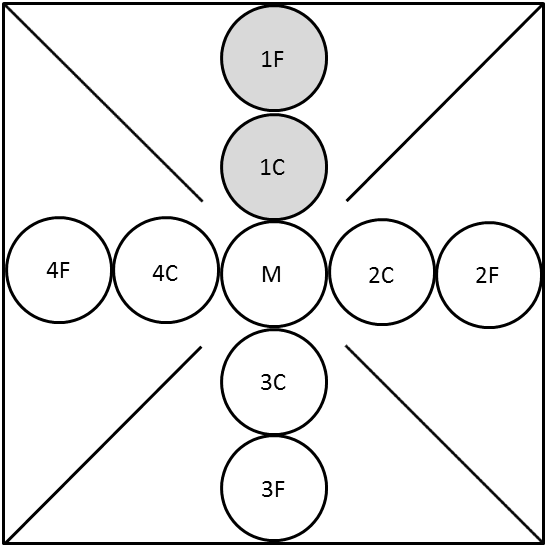


Fig. 1. Soil sampling in the pots. We pushed two-cm-diameter soil corers into the soil until the bottom of the pot. We took one core in the middle (M), four cores at the side of the pot (F) and four in the middle (C). We numbered the four quarters clockwise in which the high nutrient quarter was labeled as 1.

Fig. 2. The nitrogen concentration of the soil solution (μg N (NO­­­­_2_^-^ and NO_3_^-^)/ g dry soil) in the different quarters (1-4) and the middle (M) of the pots (see Fig. 1) during the course of the experiment. Samples were taken just before (open symbols) or just after fertilization (closed symbols) in the first (July 22^nd^), third (August 8^th^) and fifth (August 22^nd^) week of the experiment.
